# Increased soil moisture aggravated the competitive effects of the invasive tree *Rhus typhina* on the native tree *Cotinus coggygria*

**DOI:** 10.1186/s12898-020-00284-9

**Published:** 2020-03-30

**Authors:** Xiao Guo, Zhen-Wei Xu, Ming-Yan Li, Xiao-Huang Ren, Jian Liu, Wei-Hua Guo

**Affiliations:** 1 College of Landscape Architecture and Forestry, Qingdao Agricultural University, Qingdao, 266109 People’s Republic of China; 2 Institute of Ecology and Biodiversity, College of Life Sciences, Shandong University, Qingdao, 266237 People’s Republic of China; 3 Shandong Provincial Engineering and Technology Research Center for Vegetation Ecology, Shandong University, Qingdao, 266237 People’s Republic of China; 4 Institute of Environmental Research, Shandong University, Qingdao, 266237 People’s Republic of China

**Keywords:** Competition, Biomass allocation, Photosynthesis, Plant invasion, Water gradient

## Abstract

**Background:**

Invasive exotic species have caused significant problems, and the effects of extreme precipitation and drought, which might occur more frequently under the global climate change scenarios, on interspecific relationship between invasive and native species remain unclear.

**Results:**

We conducted a greenhouse experiment with three soil water levels (30–40%, 50–60%, and 70–80% of field capacity) and two cultivation treatments (monoculture pots, one seedling of either species and mixture pots, one seedling of each species) to investigate soil water content effects on the relationship between invasive *Rhus typhina* and native *Cotinus coggygria*. *Rhus typhina* had lower height but bigger crown area than *C. coggygria* in the monoculture treatment. *Rhus typhina* had higher height, bigger crown area and total biomass than *C. coggygria* in the mixture treatment. Drought decreased the growth parameters, total chlorophyll concentration, and leaf biomass, but did not change gas exchange and other biomass parameters in *R. typhina*. The growth parameters, leaf area index, biomass parameters, total chlorophyll concentration, and net photosynthetic rate of *C. coggygria* decreased under drought conditions. The log response ratio (lnRR), calculated as ln (total biomass of a target plant grown in monoculture/total biomass of a target plant grown in mixed culture), of *R. typhina* was lower than that of *C. coggygria*. The lnRR of *R. typhina* and *C. coggygria* decreased and increased with increase in soil water content, respectively.

**Conclusions:**

*Rhus typhina* has greater capacity to relatively stable growth to the drought condition than *C. coggygria* and has strong competition advantages in the mixture with *C. coggygria*, especially in the drought condition. Our study will help understand the causes of invasiveness and wide distribution of *R. typhina* under various moisture conditions and predict its expansion under climate change scenarios.

## Background

The global climate change has resulted in an increasing trend in extreme precipitation in some areas, and severe drought is predicted to occur more frequently in the future in several regions of the world [1]. Water limitation is one of the main factors limiting plant growth and is the most general type of stress experienced by plants [2]. Drought restrains eco-physiological performances of plants, such as photosynthesis and primary productivity [3, 4] and changes the community structure [5], creating an opportunity for the invasion of exotic species, most likely leading to in the decline of biodiversity [6] and instability of the entire ecosystem [7, 8].

Identifying the determinants of the invasiveness of alien plant species is essential in invasion ecology, and it has considerable implications [9]. It has been reported that the invasive species always have faster growth rate, stronger photosynthesis [10], and more plastic biomass allocation [11] than those of native species [12]. These differences can be explained by the phenotypic divergence hypothesis [13, 14], which suggests that the higher the difference in functional traits between the invasive and native species, the higher the probability of successful invasion. However, based on the process of habitat filtering, phenotypic convergence hypothesis [15, 16] states the importance of environmental factors and holds that invasive and coexisting native species have a strong similarity in functional traits. This strong similarity confers the exotic species the ability to adapt to local environments and to invade native communities more easily [15, 17]. The phenotypic convergence hypothesis has been supported by some empirical studies [18, 19]. Despite numerous studies, there is no consensus on this hypothesis.

The competition for resources is a crucial process that influences plant competitive interactions and invasiveness [6, 9, 20]. According to the theory of fluctuating resource availability [21], increased resources will increase probability of invasion success of invaders into a plant community. In addition, native species have been reported to outperform the invasive species under limited resource conditions [17]. However, it is not always the case as invasive species were more or similar efficient to acquire resources compared to native species under conditions of low resources [22–26]. Invasive plant can change the leaf traits to adapt the shade environment, such as expanding leaf area to increase light acquirement [27]. Besides, if the growth of exotic and native species is restricted by different resources, the increase in certain resources might not exacerbate the competitive effects of exotics [28, 29]. Therefore, the consistent conclusion is still unclear.

Water plays an important role in the growth and physiology of plants and significantly affects the interaction between native and invasive species [30]. Drier soils have been demonstrated to significantly increase the total biomass of invasive species, whereas, the native species are unaffected by the soil water status [31]. Invasive plant can adjust the resource allocation to leaf, which can decrease leaf are to reduce transpiration loss [32]. However, the relative growth rate of invasive *Lantana camara* decreased under drought condition; thus, limiting its invasion into arid and sub-arid environments [33]. Furthermore, some studies found that native species is a better competitor than invasive species, regardless of the water regime [34] or water supply pattern [35]. Experimental evidence is still insufficient and the effect of soil moisture on the competitive effects of invasive species remains unclear.

To examine the effects of invasive species on native species in response to various water conditions, we conducted a greenhouse experiment with an invasive species (*Rhus typhina*) and a confamiliar native species (*Cotinus coggygria*). *Rhus typhina*, a large shrub or small tree native to eastern North America [36], is highly invasive in Europe [37], and it is also regarded as an invader in China [38]. The species can propagate through root suckers to occupy the territory rapidly, and it can tolerate extremely poor soils with rapid reproduction and vigorous growth [39]. *Cotinus coggygria* is widely distributed in the warm-temperate zone of China by local adaptation [40], and it tends to be a small tree or a shrub [41, 42]. Both the species are regarded as ornamental plants, and they are known for their red autumnal leaves [39, 42]. *R. typhina* and *C. coggygria* exist in the temperate deciduous forest in the eastern Asia and have strong competition with each other, especially at the stage and have similar ecological niche at the stage of seedlings and mature trees. Besides, the two species are widely used for reforestation in North China [39].

Most of previous studies have compared mixture treatment containing two species in one pot with monoculture treatment containing two or more single-species plants in one pot [36, 43–45]. This kind of experimental design might underestimate the effects of interspecific competition, because the comparison is made between interspecific and intraspecific competition, with intraspecific competition as control [31, 36, 46]. Therefore, in the present study, we set up a monoculture treatment with one species per pot as the control to avoid the interference of intraspecific competition. We addressed the following questions:

1. Is the invasive *R. typhina* a superior competitor in mixtures? Does *R. typhina* show convergence or divergence in functional traits relative to *C. coggygria*?
2. What functional traits confer the competitive advantage to the superior competitor?
3. Does water availability affect the competition dominance between *R. typhina* and *C. coggygria*?

## Results

### Plant growth

**Fig. 1:**
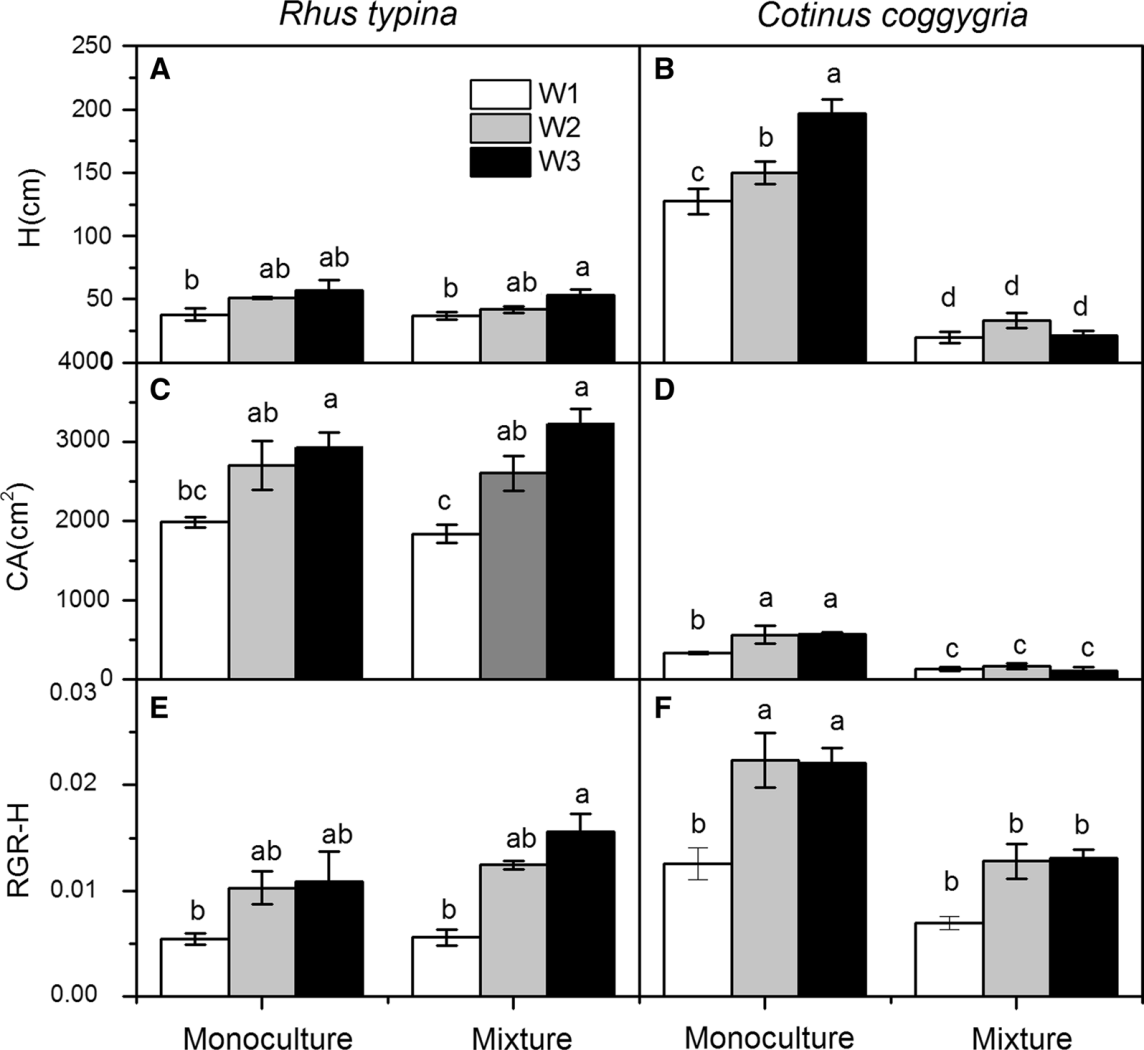
Height (H; **A**, **B**), crown area (CA; **C**, **D**), and relative growth rate for shoot height (RGR-H; **E**, **F**) of *Rhus typhina* and *Cotinus coggygria* at the end of the experiment under three water and two cultivation regimes. Data in the figure are mean ± SE (n = 5). Different letters denote significant differences (p ≤ 0.05) with Duncan’s method

In the monoculture pots, the shoot height and RGR-H of *R. typhina* were lower than those of *C. coggygria*, whereas the crown area of *R. typhina* was higher than that of *C. coggygria* (Fig. 1). In the mixed culture pots, the shoot height and crown area of *R. typhina* were higher than those of *C. coggygria* (Fig. 1). All the growth parameters, including the shoot height, crown area, and RGR-H of *R. typhina* were generally decreased under drought condition, but not by cultivation (Table 1; Fig. 1). The interaction of drought and cultivation had no effect on any of the growth parameters of *R. typhina* (Table 1). All the growth parameters of *C. coggygria* in the monoculture pots generally decreased with decreasing soil moisture content, whereas, those of *C. coggygria* in the mixture pots were similar across all water treatments (Fig. 1).

**Table 1:** Results of two-way ANOVA for the effects of water treatments, plant cultivation and their interactions on performance and functional traits of *Rhus typhina* and *Cotinus coggygria*

	*Rhus typhina*						*Cotinus coggygria*					
	Water treatments		Plant cultivation		Interaction effects		Water treatments		Plant cultivation		Interaction effects	
	F	*p*	F	*p*	F	*p*	F	*p*	F	*p*	F	*p*
Height (cm)	*11.026*	0.00**	2.176	0.158	0.640	0.539	*9.796*	0.001**	*451.81*	0.00**	*12.927*	0.00**
Crown area (cm^2^)	*14.652*	0.00**	0.006	0.938	0.623	0.548	2.56	0.105	*57.593*	0.00**	2.491	0.111
RGR-H	*12.186*	0.000**	3.314	0.084	0.994	0.388	*14.483*	0.000**	*34.468*	0.000**	0.832	0.450
SLA (cm^2^/g)	0.706	0.508	1.872	0.189	0.916	0.419	1.635	0.221	*25.320*	0.000**	3.169	0.065
LAI	0.076	0.927	*90.586*	0.000**	1.478	0.262	3.317	0.061	*6.311*	0.022*	0.803	0.464
Chl	*4.824*	0.029*	*4.806*	0.049*	0.023	0.977	0.242	0.788	*6.182*	0.027*	1.389	0.284
Chla/Chlb	1.575	0.250	*69.498*	0.000**	*4.471*	0.038*	1.653	0.229	3.024	0.106	7.301	0.007**
A (μmol m^−2^ s^−1^)	0.161	0.853	0.011	0.918	2.133	0.161	3.010	0.087	*36.548*	0.000**	0.924	0.424
E (mmol m^−2^ s^−1^)	0.095	0.910	0.570	0.465	3.449	0.066	*3.978*	0.047*	*9.718*	0.009**	0.111	0.896
Gs (mmol m^−2^ s^−1^)	0.330	0.726	0.085	0.775	1.402	0.284	3.345	0.070	*26.780*	0.000**	0.322	0.731
Ci (mmol mol^−1^)	0.243	0.788	0.501	0.492	0.667	0.531	0.554	0.589	3.196	0.099	0.189	0.830
WUE	0.438	0.655	*5.854*	0.032*	*6.946*	0.01*	1.714	0.221	*12.42*	0.004*	2.482	0.125
Total biomass (g)	1.095	0.355	0.205	0.655	1.531	0.242	*26.759*	0.000**	*424.422*	0.000**	*26.484*	0.000**
Leaf biomass (g)	*7.636*	0.004**	0.446	0.512	2.252	0.133	*13.846*	0.000**	*299.123*	0.000**	*12.491*	0.000**
Stem biomass (g)	0.517	0.605	0.003	0.958	1.213	0.319	*19.317*	0.000**	*535.269*	0.000**	*19.453*	0.000**
Root biomass (g)	1.708	0.208	1.655	0.214	1.903	0.176	*4.100*	0.034*	*201.801*	0.000**	*3.927*	0.038*
Root-shoot ratio	2.555	0.104	2.997	0.100	1.468	0.255	2.015	0.162	4.179	0.056	0.554	0.584
Leaf biomass ratio	2.398	0.118	0.100	0.755	0.136	0.874	1.039	0.374	*26.198*	0.000**	3.302	0.060
Stem biomass ratio	0.371	0.695	1.584	0.223	0.770	0.477	2.447	0.115	*127.233*	0.000**	*3.689*	0.045*
Root biomass ratio	1.815	0.190	3.074	0.096	1.685	0.212	2.030	0.160	*4.622*	0.045*	0.544	0.590

*RGR-H* relative growth rate of height, *LAI* leaf area index, *SLA* specific leaf area, *Chl* total chlorophyll concentration, *Chla/Chlb* chlorophyll a to b ratio, *A* maximum net photosynthetic rate, *E* transpiration rate, *Gs* stomatal conductance, *Ci* Intercellular carbon dioxide concentration, *WUE* water use efficiency

Significant effects are indicated by italic and asterisks: **p < 0.01, *0.01 < *p* ≤ 0.05

### Leaf traits

**Fig. 2:**
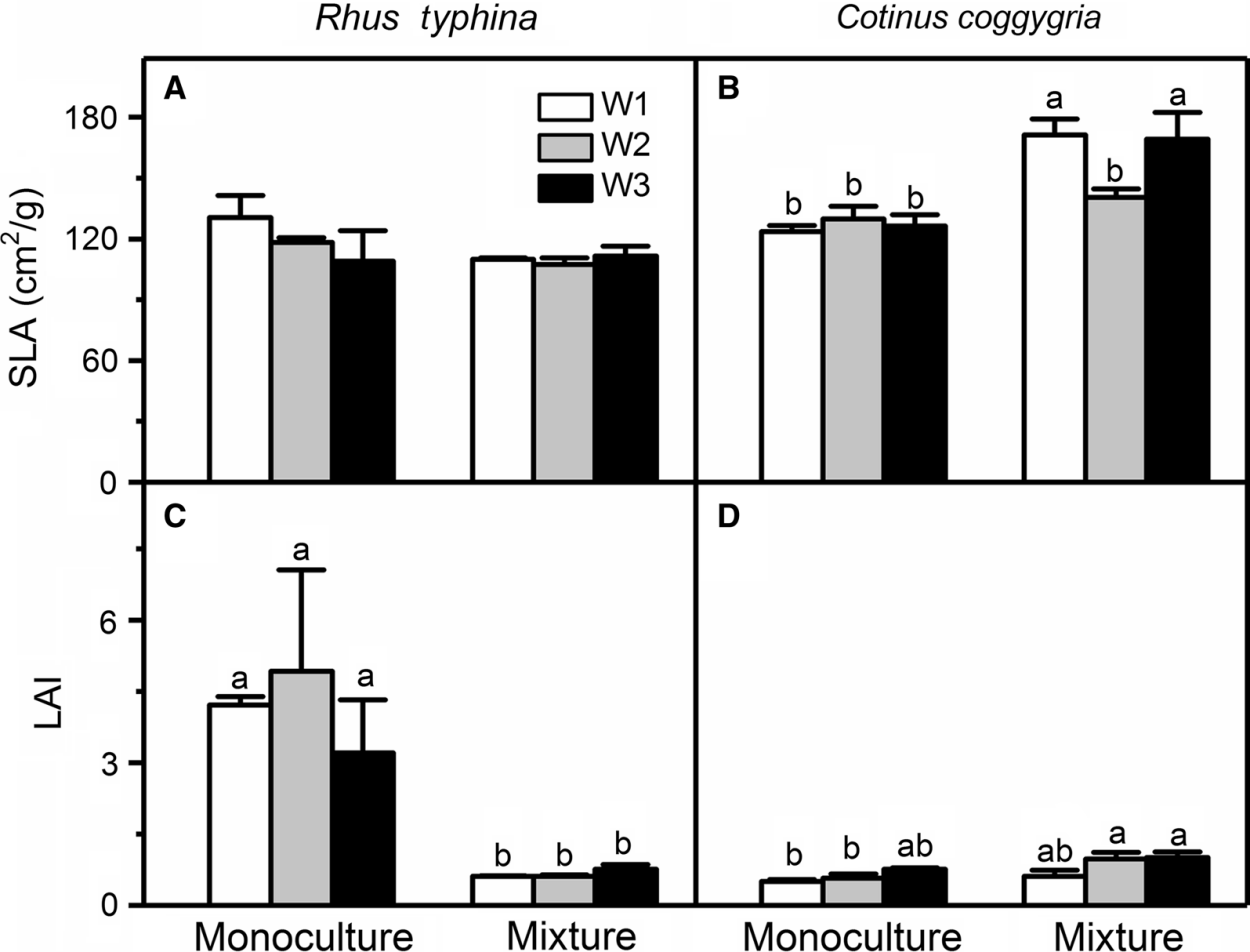
Specific leaf area (**A**, **B**) and leaf area index (LAI; **C**, **D**) of *Rhus typhina* and *Cotinus coggygria* under three water and two cultivation regimes. Data in the figure are mean ± SE (n = 5). Different letters denote significant differences (p ≤ 0.05) with Duncan’s method

In the monoculture pots, the LAI of *R. typhina* was higher than that of *C. coggygria* (Fig. 2). In the mixture pots, the SLA of *R. typhina* was lower than that of *C. coggygria* (Fig. 2). Water had no effect on the SLA and LAI of the two species (Table 1). Furthermore, the SLA of *R. typhina* was not affected by cultivation treatment as well. The SLA and LAI of *C. coggygria* generally increased in the mixture pots, compared with those of *C. coggygria* in the monoculture pots (Table 1; Fig. 2B, D). On the contrary, the LAI of *R. typhina* was decreased by mixed culture (Table 1, Fig. 2A).

### Biomass and allocation

In the monoculture pots, the total biomass and leaf biomass of *R. typhina* were higher than those of *C. coggygria* under W1 (30–40% of field capacity) and W2 (50–60% of field capacity) conditions, but lower than those of *C. coggygria* under W3 (70–80% of field capacity) condition. The root biomass of *R. typhina* was higher than that of *C. coggygria* in all the water treatments. In the mixture pots, the total biomass, leaf biomass, stem biomass, and root biomass of *R. typhina* were higher than those of *C. coggygria* in all the water treatments.

**Fig. 3:**
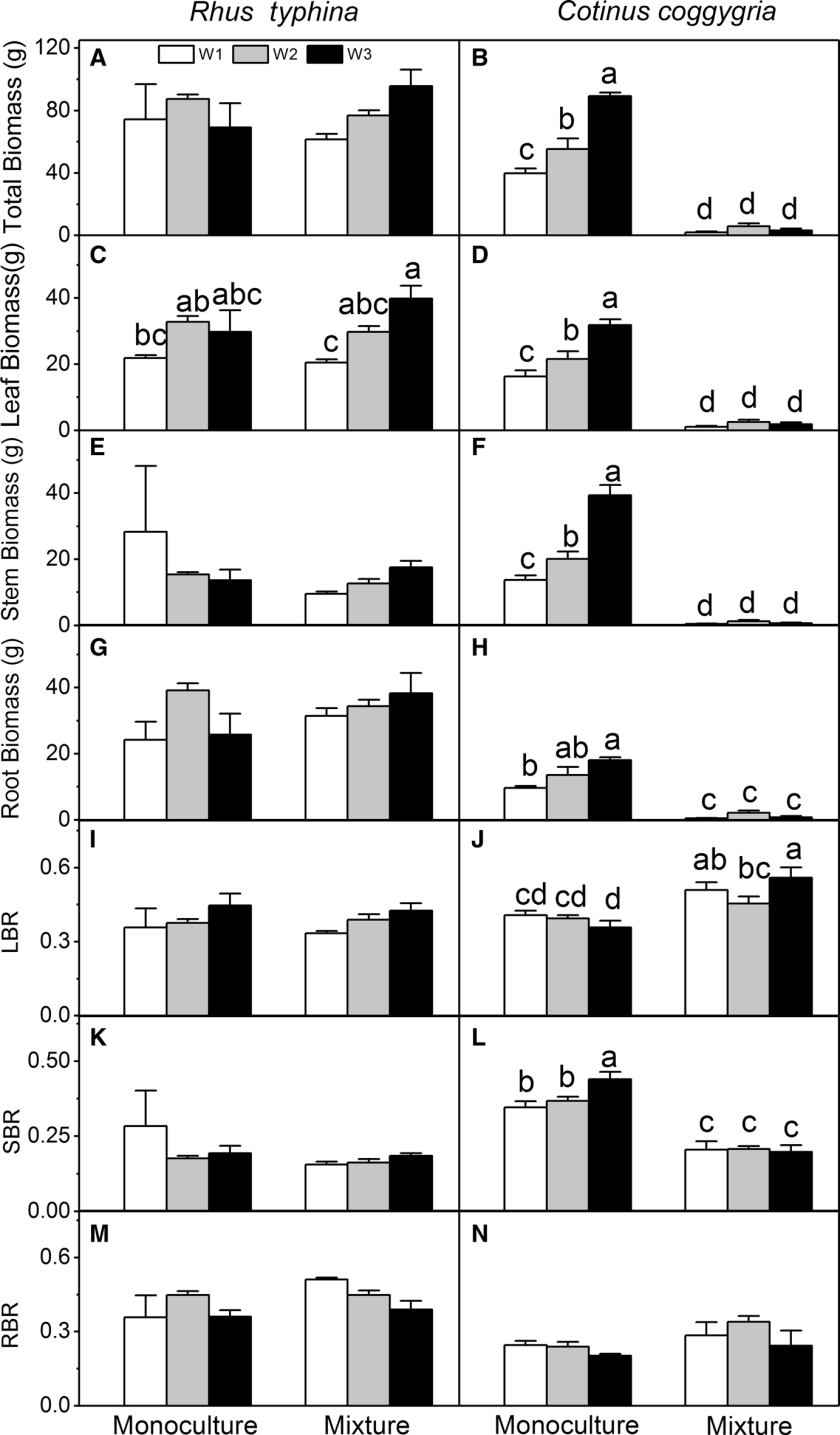
Biomass parameters and biomass allocation parameters of *Rhus typhina* and *Cotinus coggygria* at the end of the experiment under three water and two cultivation regimes. The biomass parameters include total biomass (**A**, **B**), leaf biomass (**C**, **D**), stem biomass (**E**, **F**), and root biomass (**G**, **H**). The biomass allocation parameters include leaf biomass ratio (LBR; **I**, **J**), stem biomass ratio (SBR; **K**, **L**) and root biomass ratio (RBR; **M**, **N**). Data in the figure are mean ± SE (n = 5). Different letters denote significant differences (p ≤ 0.05) with Duncan’s method

In *R. typhina*, neither cultivation nor interaction between cultivation and water affected any of the biomass and allocation parameters and water affected only the leaf biomass (Table 1). The biomass parameters of *C. coggygria* were affected by cultivation and water, as well as their interaction. Mixed culture decreased all the biomass parameters and the SBR of *C. coggygria*; the LBR and RBR increased in the mixture pots compared with those in the monoculture pots (Table 1, Fig. 3). Drought generally decreased the leaf biomass of *R. typhina* in both the cultivation treatments (Table 1; Fig. 3C). Drought decreased all the biomass parameters of *C. coggygria* in the monoculture pots, whereas, those of *C. coggygria* in the mixture pots were not affected (Fig. 3).

### Chlorophyll and photosynthetic characters

**Fig. 4:**
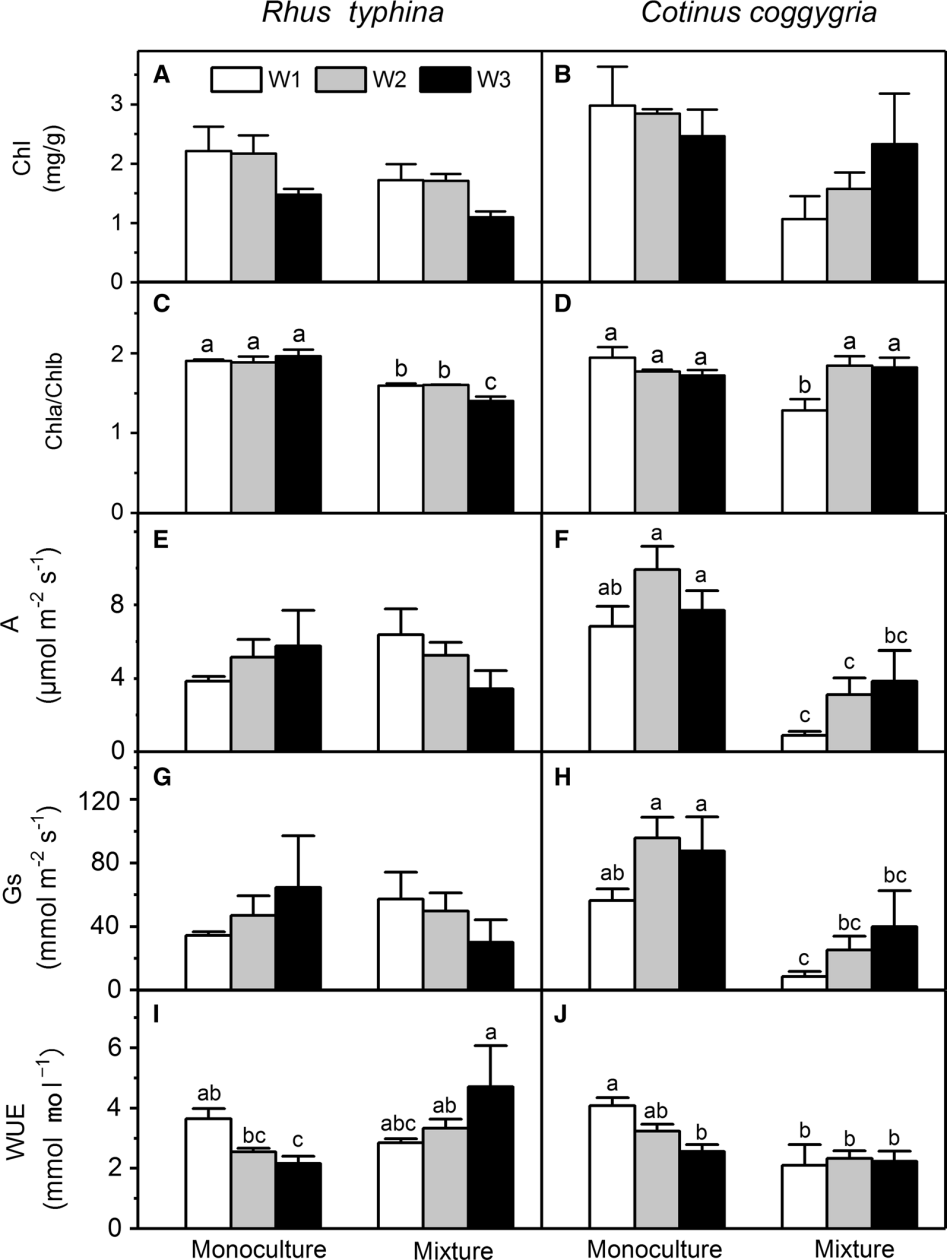
Chlorophyll parameters and photosynthesis parameters of *Rhus typhina* and *Cotinus coggygria* under three water and two cultivation regimes. The chlorophyll parameters include chlorophyll concentration (chl; **A**, **B**) and chlorophyll a to chlorophyll b ratio (chla/chlb; **C**, **D**). The photosynthesis parameters include net photosynthesis rate (A; **E**, **F**), stomatal conductance (Gs; **G**, **H**) and water use efficiency (WUE; **I**, **J**). Data in the figure are mean ± SE (n = 3). Different letters denote significant differences (p ≤ 0.05) with Duncan’s method

In the pots, the total chlorophyll concentration, A, and Gs of *R. typhina* was lower than those of *C. coggygria* (Fig. 4). In the mixture pots, the total chlorophyll concentration of *R. typhina* was lower than that of *C. coggygria* under W3 condition but higher than that under W1 and W2 conditions (Fig. 4). In the mixture pots, the A and Gs of *R. typhina* were higher than that of *C. coggygria* under W1 and W2 conditions but were similar to those of *C. coggygria* under W3 condition. In the monoculture pots, the WUE of *R. typhina* was similar to that *C. coggygria* but was higher than that of *C. coggygria* in all the water treatments in mixture pots (Fig. 4). The WUE of *R. typhina* increased in the mixed culture compared to that in the monoculture, whereas the WUE of *C. coggygria* in the mixed culture decreased compared to that in the monoculture (Fig. 4).

The total chlorophyll concentration of *R. typhina* was decreased by increasing soil water content and mixed culture (Table 1, Fig. 4). The total chlorophyll concentration of *C. coggygria* was decreased by mixed culture (Table 1, Fig. 4). The chlorophyll a to chlorophyll b ratio of *R. typhina* was decreased by mixed culture (Table 1, Fig. 4). The interaction between mixed culture and water affected the chlorophyll a to chlorophyll b ratio in *R. typhina* (Table 1). However, neither mixed culture nor water had effect on the chlorophyll a to chlorophyll b ratio of *C. coggygria* (Table 1). Plant cultivation and soil water content had no effect on the A of *R. typhina*, whereas mixed culture decreased that of *C. coggygria* (Table 1; Fig. 4). Mixed culture increased the WUE of *R. typhina*, but decreased that of *C. coggygria* (Table 1; Fig. 4).

### Competitive interaction between two species

**Fig. 5:**
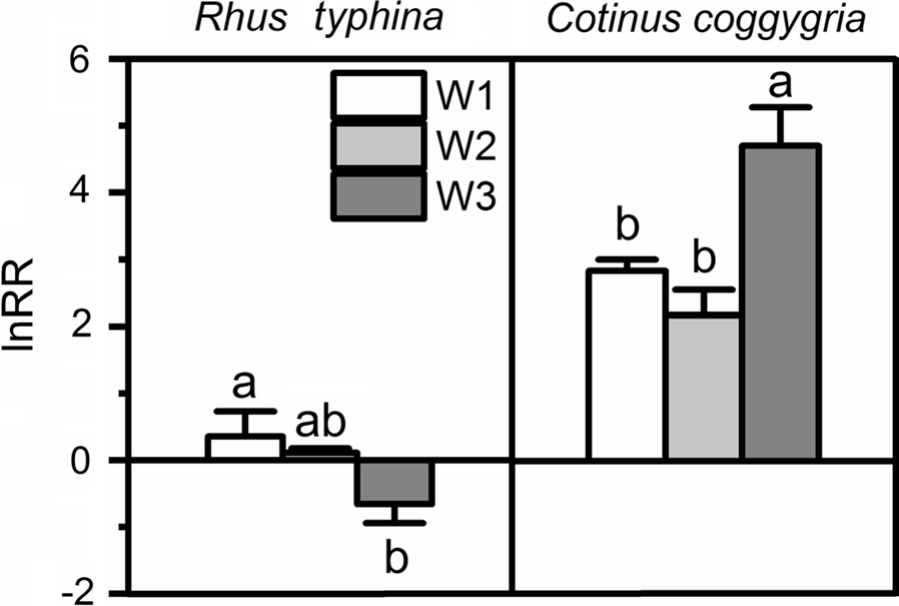
The log response ratio (lnRR) of *Rhus typhina* and *Cotinus coggygria* under three water regimes. The lnRR was calculated as ln (total biomass of a target plant grown in monoculture/total biomass of a target plant grown in mixed culture). Data in the figure are mean ± SE (n = 5). Different letters denote significant differences (p ≤ 0.05) with Duncan’s method

The lnRR of *R. typhina* decreased with increase in soil moisture content, whereas, that of *C. coggygria* increased (Fig. 5). The lnRR of *R. typhina* was lower than that of *C. coggygria* (Fig. 5). In the W3 condition, the lnRR of *R. typhina* was less than zero, but the lnRR of *C. coggygria* was more than zero. In the W2 and W1 conditions, the lnRR of both *R. typhina* and *C. coggygria* was more than zero and the lnRR of *R. typhina* much larger than that of *C. coggygria* (Fig. 5).

## Discussion

We conducted the phylogenetic comparison to minimize phylogenetic bias as trait differences among species strongly depend on phylogenetic relationships, growth forms and life forms [9, 22, 47]. *R. typhina* has high total biomass than *C. coggygia* in the mixture treatment. This indicates the strong competitive effects of *R. typhina* on *C. coggygria* and that *R. typhina* is the superior competitor. The lnRR further validated this observation as the lnRR of *R. typhina* was always lower than that of *C. coggygria*, indicating the competitive effects on *R. typhina* was less than that on *C. coggygria*. However, we cannot conclude that mixed culture with *C. coggygria* facilitated the growth of *R. typhina* in well-watered conditions even though the lnRR of *R. typhina* was below zero under W3 condition. This is because the total biomass of *R. typhina* remained unchanged across different water regimes. *Rhus typhina* has also been reported to outcompete native *Quercus acutissima* [36] and *Vitex negundo* [35].

### Interaction between *R. typhina* and *C. coggygria* under mixed culture condition

The shifts in biomass allocation pattern of the introduced species can create a advantage for a species over their neighbors in response to different environmental conditions [17, 48]. Both *R. typhina* and *C. coggygria* had high biomass in the W3 treatment but the total biomass of *C. coggygria* rapidly decreased with the soil moisture decreasing (Fig. 3). It indicated that *R. typhina* have stronger ability of adapting the drought environment than *C. coggygria*. Previous study found that *R. typhina* have higher total biomass facing both interspecific and intraspecific pressure than native plant under both drought and wet conditions [35]. Drought may be the disturbance for plant growth and invasive plant always can adapt to disturbance condition, based on the disturbance hypothesis [49]. Drought decreased the resource allocation of *C. coggygria* to stem but had no influence on the SBR of *R. typhina* (Fig. 3), and the *R. typhina* had lower height than *C. coggygria* (Fig. 1). *R. typhina* may have stronger the wood density and increase drought tolerance. Previous study found invasive tree *Acacia mearnsii* have higher wood density and resistive capacity to drought-induced cavitation than native trees in the introduced habitat [50]. Drought changes the species composition of native plant community and make more susceptible to invasion [51].

Shoot height and allocation to growth have been shown to promote invasiveness [47]. Higher shoot height, crown area, and leaf biomass in the present study together conferred *R. typhina* a competitive advantage over *C. coggygria* to obtain more light in mixture pots. According to the productivity-dependent scaling hypothesis, high aboveground productivity of the neighboring plant will impose strong asymmetric competition on the target plant [52, 53]. This asymmetric competition for light has the strongest effect on the growth of small trees [52], i.e., *C. coggygria* seedlings in the present study, as the largest share of a vital resource can be gained by a plant through a slight height advantage over its neighbors [54]. The growth rate is probably a key functional trait linked to the invasiveness of a tree species, and it is highly associated with the invasion success of tree species [9, 47, 55]. In the mixture pots, *R. typhina* had slightly higher RGR-H and thus taller shoot to cover *C. coggygria* rapidly, suppressing the growth of the native species by shading it [14, 29]. Besides, *R. typhina* enabled to inhibit the growth of shoot adjacent native plant *Vitex negundo* var. *heterophylla* in the interspecific competition although native plant had higher height than *R. typhina* facing intraspecific competition [35].

For *C. coggygria*, the RBR increased but the root biomass decreased in mixed culture, indicating that its ability to obtain water and potential nutrients was decreased by the strong competition from *R. typhina*. Larger root biomass of *R. typhina* than that of native species were found previously [35, 36]. Furthermore, the RBR, root biomass, and WUE of *R. typhina* were higher than those of *C. coggygria* in mixed pots, which conferred *R. typhina* a conspicuous advantage to absorb more water and nutrients, and utilize the water more efficiently, hindering the growth of *C. coggygria* by decreasing its resource supply [29]. In this situation, *C. coggygria* invests more on biomass production to the root and leaf to cope with the intrusion of *R. typhina*, but this alteration in biomass allocation strategy still cannot offset the strong competitive effects from *R. typhina.* Considering the aboveground advantages mentioned above, we conclude that invasive alien species possess stronger light-capturing and water/nutrients-absorbing abilities, and water use efficiency than the native species by exhibiting more acquisitive functional traits. Previous found that facing the pressure of intraspecific competition, the *R. typhina* have higher total biomass and higher root biomass ratio to increasing the absorption nutrient than native plant *Quercus acutissima* Carr. and this advantage is also maintained during interspecies competition [36].

This conferred *R. typhina* an assimilatory advantage over *C. coggygria*; thus, contributing to higher performance than that of *C. coggygria* in the mixture pot. Higher photosynthetic traits are considered typical features for the success of invasive species [9, 14, 36, 47] and may be the reason for the higher growth rate of shoot height of *R. typhina* in the present study. *Cotinus coggygria* seedlings tend to grow upward (higher shoot height, RGR-H, and SBR than those of *R. typhina*) to reach the upper space with higher maximum net photosynthetic rate (A) than that of *R. typhina*. *Rhus typhina* seedlings tend to grow horizontally (higher crown area than that of *C. coggygria*) to capture more light and deeply to obtain more water and nutrient (higher root biomass and RBR than those of *C. coggygria*). *Rhus typhina* maintained larger crown area, leaf biomass, and root biomass than those of *C. coggygria*. The upward growth of *C. coggygria* seedlings was strongly inhibited (indicated by distinctly lower shoot height than that of *C. coggygria* in monoculture) and its A was decreased most likely due to the shade environment caused by the exceedingly larger canopy of *R. typhina* seedlings in the upper layer in the mixture. Eventually the total biomass of *C. coggygria* seedlings was extremely lower than that of *R. typhina* seedlings in the mixture pots. Therefore, the invasive *R. typhina* and the native *C. coggygria* displayed differences in most crucial functional parameters related to growth when they were cultivated separately or together, indicating that our results support the phenotypic divergence hypothesis [13] rather than the phenotypic convergence hypothesis [15, 16].

Invasive alien *R. typhina* possesses three advantages over the native *C. coggygria* in the mixed culture. First, with the stronger photosynthetic capacity than *C. coggygria*, *R. typhina* was able to achieve a higher growth rate and larger amount of carbohydrate production. Secondly, in the mixture pots, higher shoot height, crown area, RGR-H, and leaf biomass enabled *R. typhina* to exhibit dominance earlier by obtaining more light resource and shading the native *C. coggygria*. This asymmetric competition caused by the higher aboveground productivity of *R. typhina* over *C. coggygria* results in positive-feedback [53] to *R. typhina*, leading to even higher competitive advantages of *R. typhina*. Finally, the higher root biomass and WUE of *R. typhina* than *C. coggygria* conferred *R. typhina* the ability to obtain water and nutrients, and utilize water more efficiently.

### Drought relieves the competitive effects of *R. typhina* on *C. coggygria*

*Rhus typhina* maintained its dominance under drought condition despite the fact that the competitive ability of *R. typhina* over *C. coggygria* decreased and concomitantly the competitive ability of *C. coggygria* over *R. typhina* increased to some extent. This observation generally supports the fluctuating resource availability theory [21], which holds that a plant community’s invasibility decreases under limited resource conditions. The decrease in the competitive ability of *R. typhina* over *C. coggygria* under drought conditions was probably related to the growth inhibition of *R. typhina* (i.e., decrease in the shoot height, crown area, RGR-H, and leaf biomass) under drought conditions. However, the shoot height, crown area, RGR-H, and leaf biomass of *R. typhina* were still substantially higher than those of *C. coggygria* under drought condition, consistently conferring *R. typhina* an aboveground competitive advantage. Therefore, the asymmetric competition of *R. typhina* over *C. coggygria* was alleviated to some extent, because the light-capturing ability of *R. typhina* decreased to some extent, although this ability was still conspicuously stronger than that of *C. coggygria*. *Rhus typhina* has been shown to outperform native *Vitex negundo* under variable levels of water supply frequency but with a constant level of total supplied water [35].

Drought decreased all the biomass parameters of *C. coggygria* in the monoculture pots, whereas, those in the mixture pots was unaffected by drought, suggesting that the mixed culture alleviated the negative effects of drought on *C. coggygria*. This might be because *R. typhina* has a larger crown area than that of *C. coggygria*, which would intercept more light, reduce the temperature of the topsoil and evaporation of soil water, and eventually alleviate the negative effects of drought on *C. coggygria* when it is grown close to *R. typhina*. It has been reported that shade can alleviate the negative effects of drought on *Acer buergerianum* [56], which is consistent with our results. The decreased chlorophyll a to chlorophyll b ratio of *C. coggygria* under drought conditions provided further evidence to this view, because decreased chlorophyll a to chlorophyll b ratio enables the plant to obtain more light in shade [56]. Therefore, the increase in the competitive ability of *C. coggygria* over *R. typhina* under drought condition was likely caused by weaker drought effects on *C. coggygria* in the mixture pots compared with that in the monoculture pots. In mixture pots, the root biomass and RBR of both species remained unchanged with the increasing drought stress, and *R. typhina* had higher root biomass, RBR, and WUE than those of *C. coggygria*, indicating that the belowground advantage of *R. typhina* to obtain water and nutrients, and utilize water more effectively than *C. coggygria* was independent of soil water content.

The A and Gs (stomatal conductance) of *C. coggygria* simultaneously decreased by both drought and the competition with *R. typhina*, indicating the stomatal closure of *C. coggygria* in response to drought and competition. The responses of *C. coggygria* in the present study support the carbon-starvation hypothesis [57, 58], in which a plant reduces Gs as the soil water potential decreases, decreasing photosynthetic carbon uptake; thus, resulting in lower biomass compared with that under other water treatment conditions in monoculture and mixed culture. Carbohydrate reserves deplete due to continued demand for carbohydrates in order to maintain metabolism, and the plant might die due to its inability to resist attack from biotic agents or starvation, whichever occurs first [58]. The stomatal conductance and net photosynthesis rate of *Acer platanoides* and *Fagus sylvatica* were found to decrease simultaneously under drought plus competition conditions [30], which is consistent with that observed in *C. coggygria* in the present study. In contrast to *C. coggygria*, *R. typhina* maintained constant A, and Gs in the mixture pots, regardless of the water conditions, indicating that *R. typhina* might have adopted another mechanism to cope with drought. Some species maintain positive carbon gain under drought conditions, allowing the midday leaf water potential to decline [57, 58] by keeping the stoma open. In this case, *R. typhina* continued to accumulate carbohydrate under drought condition, maintaining the biomass constant compared with that under well-watered condition [57], and they can withstand prolonged drought condition before carbon starvation [58].

The invasive *R. typhina* and the native *C. coggygria* displayed differences in many growth-related parameters when they were cultivated either separately or together. This is consistent with the phenotypic divergence hypothesis [13] holding that the functional traits differences contribute to the invasion success of exotic species. *Rhus typhina* maintained its absolute dominance against *C. coggygria* in both well-watered and drought environments by possessing higher photosynthetic capacity, larger crown area, taller shoot height, and higher RGR-H, leaf and root biomass, and WUE, outcompeting *C. coggygria* vertically and horizontally. The growth of *C. coggygria* was strongly limited by the competition from *R. typhina*; however, this competition alleviated the negative effects of drought on *C. coggygria* mainly by providing shade environment. Drought alleviated the asymmetric competition of *R. typhina* over *C. coggygria* to some extent because drought had stronger negative effects on than on *C. coggygria* in the mixture pots and *R. typhina* is better capable of exploiting excess water resource. Our observation provides further evidence for the fluctuating resource availability theory [21]. Under drought conditions, *C. coggygria* suffered from carbon starvation, whereas, *R. typhina* retained the normal carbohydrate synthesis, contributing to the dominance of *R. typhina*. However, both the species would experience the risk of hydraulic failure under severe drought conditions. Our observation confirmed the robustness of the comparative trait differences between invasive and non-invasive species across environmental gradients [9].

## Conclusions

*Rhus typhina* grows slowly at the seedling stage, but grows fast once established [59]. Therefore, *R. typhina* probably will continue its dominance when grown adjacent to *C. coggygria* in a long term. As the glasshouse study cannot simulate the complex natural environments, our observation should not be extrapolated arbitrarily to field conditions. Given the widely acknowledged invasiveness of *R. typhina* and the extensive use of the two species in reforestation and urban greening, field studies with closer attention on the interaction between *R. typhina* and *C. coggygria* in the future global change scenarios is necessary to better understand the invasion of *R. typhina* and to take appropriate precautionary and management measures. Our study contributes to a better understanding of invasive mechanisms of alien tree species such as *R. typhina* under various moisture conditions and predicts its future expansion under global climate change scenarios.

## Methods

### Study site and plant materials

The experiment was conducted in the greenhouse of Fanggan Research Station of Shandong University (36°26′ N, 117°27′ E), which is located in the central mountainous region of Shandong Province, China. The region has a typical temperate monsoon climate, with an average annual precipitation of approximately 600–800 mm, most of which occurs from June to August (60–70%), which was collected from National Climate Center of China in 2011. The predominant vegetation in this area is mixed forests of the warm temperature zone. The soil is a yellow cinnamon soil with limestone as the parent material [60].

The seeds of both species were collected from the Loahu hill (36°43′ N, 117°47′ E) near the research station during the October of 2011.The seeds were stored at 0–4 °C during winter in the fridge (BC-50ES, Haier Company, China). During the late April of 2012, the seeds were soaked in distilled water for 24 h before they were allowed to germinate in a growth chamber (PRX-1500C-LED, Tianlin Technology Co., Ltd, China). A voucher specimen of this material has been deposited in the herbarium of Shandong University. Healthy and uniformly germinated seedlings were transplanted into plastic pots (320 mm height × 290 mm diameter) with one or two seedling(s) per pot. Each pot was filled with 6 kg of loam and 2 kg of sand, which was carefully passed through a 2-mm sieve to remove debris, and thoroughly mixed.

### Experimental design

Three water treatments, 30–40% (W1), 50–60% (W2), and 70–80% (W3) of field capacity (FC), were applied to three plant cultivation treatments. The plant cultivation treatments included monoculture pots that contained one seedling of either species (*R. typhina* or *C. coggygria*) and mixture pots that contained one seedling of both the species.

The pots were randomly arranged in the greenhouse and re-randomized at regular intervals (7 days) throughout the experiment. The pots received compensatory irrigation after weighing daily at 18:00 h to maintain constant soil moisture level. Insects and weeds were controlled manually. Each treatment contained eight pots. The study was carried out from July 6 to September 5 of 2012, and it lasted for 62 days.

### Measurement of plant traits

The gas exchange parameters were measured on August 20 and 21, 2012, using a portable leaf gas exchange system (GFS-3000; WalzGmbH, Effeltrich, Germany). Light was supplied by a red-blue light-emitting diode set at an irradiance of 2000 µmol m^−2^ s^−1^ (PAR) to ensure that all seedlings were light saturated. The airflow through the leaf chamber was set to 400 µmol^−1^ s ^−1^, chamber temperature to ambient temperature, and CO_2_ concentration to 400 µmol mol^−1^ (Ca). The net photosynthetic rate (A), stomata conductance (Gs), transpiration rate (E), and intercellular CO_2_ concentration (Ci) were recorded. The water use efficiency (WUE) was calculated as A/E. Three healthy and fully expanded leaves on the upper shoot of three seedlings were used to measure the parameter in each treatment.

The concentration of chlorophyll was determined on August 24 and 25, 2012, according to a previous study [61], using a UV-2100 spectrophotometer (Unico, Shanghai, China). Six healthy and fully expanded leaves on the upper shoot of three seedlings (two leaves per seedling) per treatment were collected to determine the chlorophyll concentration.

The shoot height and crown area were measured at the end of the experiment. The crown area was calculated as: crown area = π a b (where, a and b are the lengths of diagonal). Five pots were selected to measure the shoot height and crown area. The relative growth rate of shoot height (RGR-H) was calculated as: RGR-H = (InH2 − InH1)/Δt; where, H2 and H1 are the shoot height values measured at the end and the beginning of the treatment, and Δt is the time duration.

Five pots per treatment were harvested thereafter, washed thoroughly with tap water, and divided into stems, leaves, and roots. The leaf area of all the plants was measured using a scanner (Epson Perfection V700; Seiko Epson, Japan) and calculated using an image analyzer (Image Pro Plus Version 4.5; Media Cybernetic, Inc., Silver Spring, MD, USA). The dry weight of various plant fractions was determined after drying in an oven for 48 h at 100 °C. The specific area (leaf area/leaf biomass, SLA), leaf area index (leaf area/crown area, LAI), root biomass ratio (root biomass/total biomass, RBR), stem biomass ratio (stem biomass/total biomass, SBR), and leaf biomass ratio (leaf biomass/total biomass, LBR) were calculated thereafter.

### Statistical analyses

To evaluate the major effects and interactions of water and cultivation treatments, the two-way analysis of variance (ANOVA) was performed using plant performance and functional traits of each species. The data were log-transformed when necessary to meet the normality and homogeneity of variance assumptions of ANOVA. However, for clarity the untransformed data are presented. The two-way ANOVA and Duncan’s multiple range test (DMRT) at 95% confidence level for all plant variables were conducted.

The dominance of the target species in mixed cultures was estimated using the log response ratio (lnRR) [20, 62, 63], calculated using the following formula: lnRR = ln (total biomass of a target plant grown in monoculture/total biomass of a target plant grown in mixed culture). The species lnRR analyzed by the one-way ANOVA was expressed separately for each species.

All the statistical analyses were performed using the IBM SPSS 21.0 software package (IBM Inc., Armonk, NY, USA). Bar charts were drawn using Origin 9.0 software (OriginLab Co., Northampton, MA, USA).

## Data Availability

All data generated or analyzed during this study are included in this published article. The datasets in this study are available from the corresponding author on reasonable request.

## Abbreviations

RGR-H: Relative growth rate of height
LAI: Leaf area index
SLA: Specific leaf area
Chl: Total chlorophyll concentration
Chla/Chlb: Chlorophyll a to b ratio
A: Maximum net photosynthetic rate
E: Transpiration rate
Gs: Stomatal conductance
Ci: Intercellular carbon dioxide concentration
WUE: Water use efficiency
FC: Field capacity
LAI: Leaf area index
RBR: Root biomass ratio
SBR: Stem biomass ratio
LBR: Leaf biomass ratio
lnRR: Log response ratio
